# Applications and challenges of biomarker-based predictive models in proactive health management

**DOI:** 10.3389/fpubh.2025.1633487

**Published:** 2025-08-18

**Authors:** Qiming Zhao, Chen Zhang, Wanxin Zhang, Shengchuan Zhang, Qiyuan Liu, You Guo

**Affiliations:** ^1^Medical Big Data and Bioinformatics Research Centre, First Affiliated Hospital of Gannan Medical University, Ganzhou, China; ^2^School of Public Health and Health Management, Gannan Medical University, Ganzhou, China; ^3^The First School of Clinical Medicine, Gannan Medical University, Ganzhou, China; ^4^School of Basic Medicine, Gannan Medical University, Ganzhou, China; ^5^Ganzhou Key Laboratory of Medical Big Data, Ganzhou, China

**Keywords:** proactive health management, biomarkers, predictive models, data heterogeneity, public health resources, multi-omics integration

## Abstract

Digital technology and artificial intelligence have revolutionized predictive models based on clinical data, creating opportunities for proactive health management. This review systematically evaluates the role and effectiveness of biomarker-driven predictive models across disease detection, personalized intervention, and healthcare resource optimization. Critical challenges hindering their implementation include data heterogeneity, inconsistent standardization protocols, limited generalizability across populations, high implementation costs, and substantial barriers in clinical translation. To address these challenges, we propose an integrated framework prioritizing three pillars: multi-modal data fusion, standardized governance protocols, and interpretability enhancement, systematically addressing implementation barriers from data heterogeneity to clinical adoption. This systematic approach enhances early disease screening accuracy while supporting risk stratification and precision diagnosis, particularly for chronic conditions and oncology applications. By effectively connecting biomarker discovery with practical clinical utilization, our proposed framework offers actionable methodologies that address existing limitations while guiding multidisciplinary research teams. Moving forward, expanding these predictive models to rare diseases, incorporating dynamic health indicators, strengthening integrative multi-omics approaches, conducting longitudinal cohort studies, and leveraging edge computing solutions for low-resource settings emerge as critical areas requiring innovation and exploration.

## 1 Introduction

### 1.1 Research background and objectives

Digital technology and artificial intelligence are transforming healthcare research and practice paradigms. Improved computational capabilities now enable integration of clinical testing databases, electronic health records, and multi-omics data, creating a multidimensional health ecosystem across the human lifecycle (1, 2). This multimodal data integration captures disease progression trajectories (3) and elucidates mechanisms underlying individual drug response variations through integrated analysis of pharmacogenomics and proteomics (4), creating a robust foundation for developing prognosis assessment and health risk predictive models (5).

The evolution of Artificial intelligence (AI) technologies has introduced transformative tools for medical data analysis. Deep learning algorithms, specifically, with their advanced feature learning capabilities, have enhanced the efficiency of analyzing high-dimensional heterogeneous data (6). These computational approaches systematically identify complex biomarker-disease associations that traditional statistical methods often overlook, enabling more granular risk stratification. Research demonstrates that Transformer-based algorithms enable precise disease risk stratification (7), accurate diagnostic determinations (2), and personalized treatment regimen optimization (8) through systematic identification of complex non-linear associations. These technological advances are shifting medical practice from traditional population-based approaches toward precision medicine focused on individual characteristics, with clinical efficacy validated through multicenter randomized controlled trials (9, 10).

Despite technological advances, significant challenges persist in effectively integrating biomarker data, developing reliable predictive models, and implementing these in clinical practice (11). Key challenges requiring resolution include data standardization (12), model generalizability (9), and clinical implementation pathways (13). This review systematically analyzes the application value and technical approaches of biomarker-based disease predictive models in proactive health management, while examining key challenges and corresponding strategies. Through integration of multidisciplinary perspectives from epidemiology, clinical medicine, bioinformatics, and artificial intelligence, we propose a comprehensive framework encompassing biomarker discovery, data integration, model construction, and clinical translation, providing systematic guidance for the implementation of predictive models in precision medicine.

### 1.2 The new paradigm of proactive health management

Proactive health management represents a transformative shift in modern medicine, transitioning from traditional disease diagnosis and treatment models to health maintenance approaches based on prediction and prevention (14). This transformation is grounded in the biopsychosocial medical model, emphasizing early health risk identification and implementation of targeted interventions to prevent disease onset or delay progression (5). This paradigm specifically aims to extend healthspan through preemptive interventions targeting subclinical pathological processes. Unlike traditional episodic care models, proactive systems implement continuous physiological monitoring (15) integrated with dynamic risk assessment methodologies (16), thereby maintaining functional capacity through preventive intervention. Such paradigmatic transformation aligns with strategic health initiatives, including “Healthy China 2030,” and addresses demographic challenges posed by increasing chronic disease prevalence in aging populations (17).

The evolution of this new paradigm has advanced through significant breakthroughs in clinical testing technologies, initiating a new era of biomarker research. Contemporary detection platforms (e.g., single-cell sequencing, spatial transcriptomics, and high-throughput proteomics) generate comprehensive molecular profiles including metabolomic, proteomic, and epigenetic features, offering unprecedented insights into disease mechanisms (18). Integrated profiling across these platforms captures dynamic molecular interactions between biological layers, revealing pathogenic mechanisms otherwise undetectable via single-omics approaches. This technological advancement has transformed biomarker discovery from traditional experience-based approaches to data-driven precise identification processes. For instance, the integration of multi-omics data and advanced analytical methods has improved early Alzheimer’s disease diagnosis specificity by 32%, providing a crucial intervention window (19, 20).

These technological breakthroughs have been accompanied by continuous refinement in detection methodologies, enabling cost-effective biomarker discovery and longitudinal monitoring capabilities. Progressive refinement of detection methodologies coupled with reduction in implementation costs has expanded biomarker applications beyond traditional diagnostics toward prospective risk assessment and targeted intervention strategies (21). The evolution encompasses diversification of clinical applications, transition from univariate to multivariate biomarker panels, and development of longitudinal monitoring systems capturing temporal physiological variations (22). Nevertheless, expanded application domains introduce significant methodological challenges requiring systematic resolution to realize the full potential of biomarker-driven precision health management.

### 1.3 Research methodology and content framework

To address implementation challenges and evaluate current evidence supporting biomarker-driven predictive models, we conducted a systematic literature analysis using structured review methodology. Comprehensive database searches in PubMed encompassed peer-reviewed publications from January 2020 through April 2025, employing Boolean combinations of standardized medical subject headings and field-specific terminology including “biomarkers,” “predictive models,” “health risk assessment,” “proactive health management,” and “precision medicine.” The methodological approach specifically targeted research addressing critical challenges in data heterogeneity management, model interpretability, and clinical implementation within personalized medicine contexts.

Through systematic analysis of the selected studies, the research synthesized current findings and identified key deficiencies and unresolved issues. Based on these analyses, a theoretically grounded content framework was constructed to systematically elucidate the key elements, technical pathways, and application prospects of biomarker-driven predictive models. Specifically, this review initially clarifies biomarker concepts and their fundamental role in establishing disease relationships, establishing the theoretical foundation for subsequent discussions; then explores the core value of biomarker predictive models in proactive health applications, including early risk stratification, personalized health interventions, and public health optimization; the technical framework section details the complete methodological pathway from data acquisition, biomarker screening to model construction and optimization, providing specific guidance for practical applications; finally, it analyzes systematically key challenges in data quality, model generalizability, clinical translation, and proposes corresponding solution strategies, indicating directions for future research.

Through this structured research methodology and systematic content framework, this review aims to provide researchers, clinicians, and policy makers with comprehensive knowledge regarding biomarker predictive models in proactive health management, advancing related technologies and improvements in clinical practice improvements. Particularly, we focus on analyzing from multidisciplinary perspectives the translation of biomarker predictive models into clinical practice, supporting personalized health management systems in precision medicine and facilitating medical practice transformation from passive response to proactive prevention.

## 2 Biomarker concepts and disease relationship construction

### 2.1 Basic concept definition

Biomarkers, defined by the U. S. Institute of Medicine as “objectively measurable indicators of biological processes” (23), function as indicators of normal biological processes, pathological processes, or pharmacological responses to therapeutic interventions. This definition emphasizes biomarkers’ objectivity and measurability, establishing the foundation for their clinical application.

From a molecular classification perspective, biomarkers include genetic markers, epigenetic markers, transcriptomic markers, protein markers, and metabolic markers, reflecting multi-level biological information from genes to phenotypes (24). With technological advancement, biomarker research has evolved from single molecular indicators to multidimensional marker combinations, and from static measurements to dynamic monitoring, enabling more comprehensive capture of disease biological features and providing enhanced information for precision medicine (25). Table 1 summarizes the characteristics, detection technologies, and clinical applications of major biomarker types, demonstrating their distinct values and limitations in proactive health management.

**Table 1:** Classification and clinical application characteristics of biomarkers.

Biomarker type	Molecular characteristics and origin	Detection technologies	Clinical application value	PMID
Genetic biomarkers	DNA sequence variants or gene expression regulatory changes	Whole genome sequencing, PCR, SNP arrays	Genetic disease risk assessment, drug target screening, tumor subtyping	39019673
Epigenetic biomarkers	DNA methylation, histone modifications, chromatin remodeling	Methylation arrays, ChIP-seq, ATAC-seq	Environmental exposure assessment, early cancer diagnosis, drug response prediction	37302584
Transcriptomic biomarkers	mRNA expression profiles, non-coding RNAs, alternative splicing	RNA-seq, microarrays, real-time qPCR	Molecular disease subtyping, treatment response prediction, pathological mechanism exploration	39736681
Proteomic biomarkers	Protein expression levels, post-translational modifications, functional states	Mass spectrometry, ELISA, protein arrays	Disease diagnosis, prognosis evaluation, therapeutic monitoring	37481764
Metabolomic biomarkers	Metabolite concentration profiles, metabolic pathway activities	LC–MS/MS, GC–MS, NMR	Metabolic disease screening, drug toxicity evaluation, environmental exposure monitoring	38280419
Imaging biomarkers	Anatomical structures, functional activities, molecular targets	MRI, PET-CT, ultrasound, radiomics	Disease staging, treatment response assessment, prognosis prediction	37776766
Digital biomarkers	Behavioral characteristics, physiological fluctuations, molecular sensing	Wearable devices, mobile applications, IoT sensors	Chronic disease management, health behavior monitoring, early warning	38347143

PCR, Polymerase Chain Reaction; SNP, Single Nucleotide Polymorphism; ChIP-seq, Chromatin Immunoprecipitation Sequencing; ATAC-seq, Assay for Transposase-Accessible Chromatin using Sequencing; mRNA, messenger Ribonucleic Acid; qPCR, quantitative Polymerase Chain Reaction; ELISA, Enzyme-Linked Immunosorbent Assay; LC–MS/MS, Liquid Chromatography–Tandem Mass Spectrometry; GC–MS, Gas Chromatography–Mass Spectrometry; NMR, Nuclear Magnetic Resonance; MRI, Magnetic Resonance Imaging; PET-CT, Positron Emission Tomography-Computed Tomography; IoT, Internet of Things; PMID, PubMed Identifier.

### 2.2 Establishing associations between biomarkers and diseases

**Figure 1:**
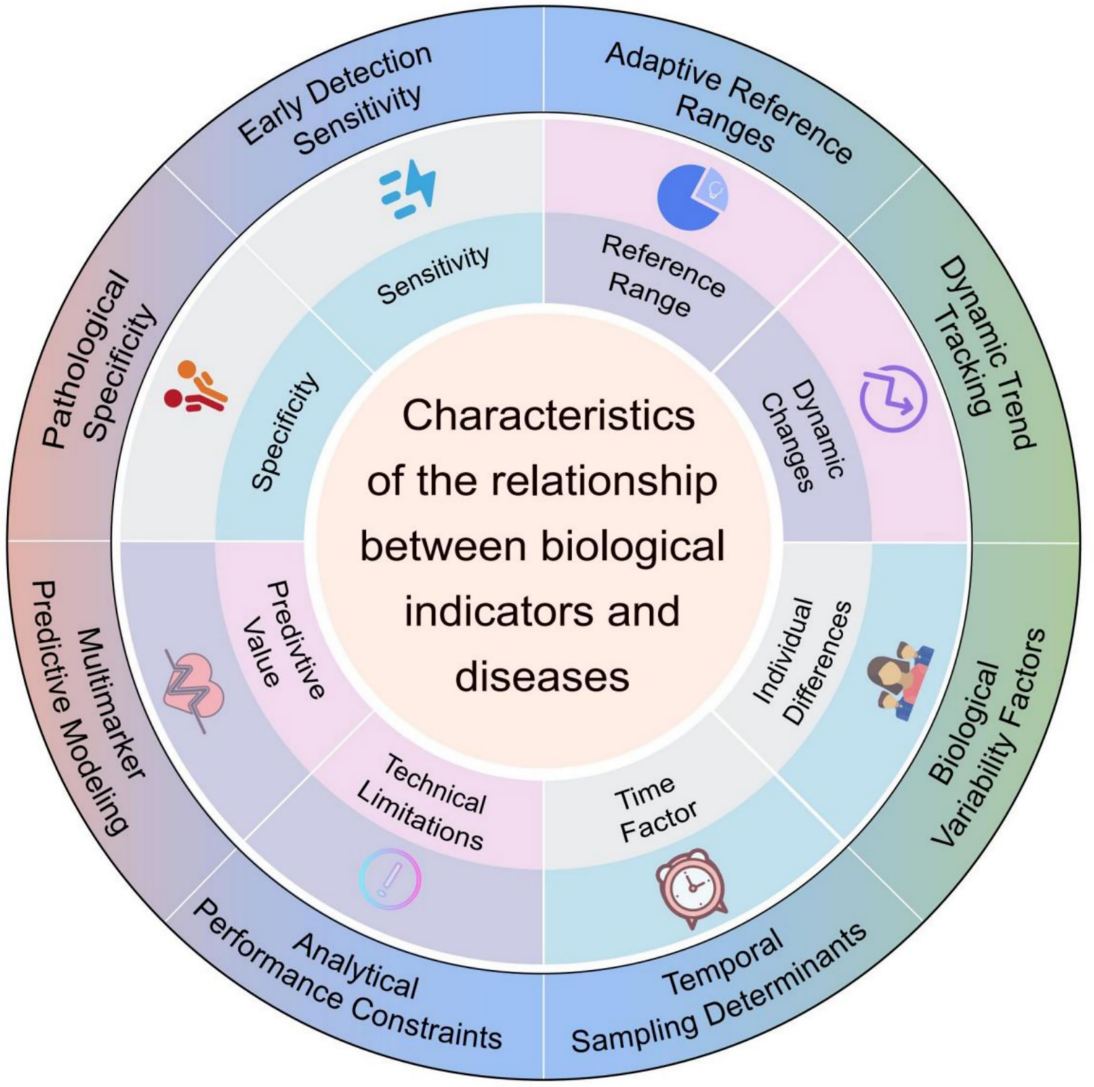
Characteristics influencing the relationship between biological indicators and diseases. The diagram outlines key factors such as sensitivity, specificity, adaptive reference ranges, dynamic changes, predictive value, technical limitations, and temporal or biological variability. These factors collectively shape the interpretive and diagnostic utility of biological indicators in disease contexts.

Establishing reliable associations between biomarkers and diseases requires integrating multidisciplinary approaches and multi-level validation. The advancement of big data and artificial intelligence technologies, has transformed biomarker research from hypothesis-driven to data-driven approaches, expanding potential marker identification (26). As illustrated in Figure 1, the relationship between biomarkers and diseases demonstrates multidimensional characteristics, including sensitivity, specificity, predictive value, dynamic changes, and technical limitations, which collectively determine their efficacy and constraints in clinical applications (27). These multidimensional characteristics collectively inform how biomarker data should be interpreted within specific clinical contexts, particularly when determining intervention thresholds that optimize the balance between diagnostic accuracy and early detection capabilities.

A systematic biomarker validation process encompasses discovery, validation, and clinical validation phases, ensuring research findings’ reliability and clinical applicability. Multi-omics integration methods serve a crucial role in this process, developing comprehensive molecular disease maps by combining genomics (5), transcriptomics (28), proteomics (29), and metabolomics data (30), thereby identifying complex marker combinations that traditional methods might overlook.

Temporal data holds distinct value in biomarker research. Through longitudinal cohort studies capturing markers’ dynamic changes over time, researchers obtain vital information about disease natural history. Studies demonstrate that marker trajectories generally provide more comprehensive predictive information than single time-point measurements (31).

### 2.3 Predictive model construction methods

Predictive model construction represents a crucial step in converting biomarker information into clinical decision-making tools. Model selection should consider data characteristics, prediction objectives, and application scenarios, aiming to a balance predictive accuracy, complexity, and interpretability (32).

Traditional statistical methods including logistic regression and Cox proportional hazards models provide strong interpretability, quantifying each marker’s contribution and enhancing clinical understanding (33). However, these approaches show limitations in managing high-dimensional data and complex non-linear relationships.

Machine learning methods such as random forests, support vector machines, and gradient boosting trees effectively process high-dimensional data, identify non-linear relationships and variable interactions, particularly excelling in multi-omics data analysis (34). Studies indicate that gradient boosting tree-based models achieve superior accuracy in cardiovascular disease risk prediction compared to traditional risk scores (35).

Deep learning shows significant potential in processing images, temporal data, and multimodal integration, though its “black box” nature restricts clinical interpretability (36). Model evaluation should thoroughly assess discriminatory power, calibration, and clinical utility, ensuring model generalizability through rigorous cross-validation and external validation. Table 2 presents a comparison of various predictive modeling methods’ advantages and limitations in processing biomarker data, offering guidance for selecting appropriate algorithms in specific health management contexts.

**Table 2:** Comparative methodology of predictive model construction.

Modeling method	Algorithm principles	Technical advantages	Limitations
Logistic/cox regression	Linear combination predicting probability or hazard ratio	Strong interpretability, computational efficiency, direct quantification of biomarker contributions	Difficulty capturing complex non-linear relationships and higher-order interactions
Random forest	Multi-decision tree voting ensemble	Strong capability for high-dimensional data, automatic identification of complex interactions, robustness to outliers	High computational resource requirements, overfitting risk, complex interpretation
Deep learning	Multi-layer neural networks extracting hierarchical features	Automatic feature extraction, multi-modal data processing, temporal feature capture	Requires large training datasets, black-box characteristics, computationally intensive
Gradient boosting trees	Tree ensemble with serial optimization of residuals	High prediction accuracy, strong capability for handling missing values, feature importance assessment	Complex hyperparameter tuning, overfitting risk, interpretability inferior to linear models
Support vector machines	Maximum margin hyperplane classification	Effective in high-dimensional spaces, kernel functions for non-linearity, strong generalization capability	Tedious parameter tuning, low computational efficiency for large datasets
Bayesian networks	Probabilistic graphical models representing causal relationships	Integration of prior knowledge, uncertainty handling, visualization of variable relationships	High computational complexity, requires expert knowledge for model structure
Ensemble methods	Multi-model combination optimizing prediction	Combines advantages of multiple models, reduces single-model risk, improves stability	Increases model complexity, reduces interpretability, increases computational overhead

In summary, biomarker predictive models demonstrate substantial application value in early disease prevention, personalized diagnosis and treatment, and public health management, advancing medical practice from reactive response toward proactive prevention. The subsequent chapter explores these models’ specific value manifestations in proactive health applications. These methodological considerations necessitate the development of an integrated technical framework, as detailed in Section 4.

## 3 Prospects for proactive health applications

Biomarker-driven predictive models present extensive application prospects in proactive health management, with their core value manifesting in three key dimensions: early risk warning, personalized health management, and public health resource optimization. These applications are progressively evolving from theoretical possibilities to clinical practice, providing robust technical support for healthcare model transformation.

### 3.1 Early disease risk warning

Predictive models based on biomarkers can detect potential health risks before clinical symptoms manifest by identifying subtle changes at molecular and cellular levels. This early warning mechanism provides valuable intervention windows for chronic disease management and tumor intervention, altering the timing and effectiveness of disease interventions (37, 38).

Multiple prospective studies validate that biomarker warning systems effectively enhance disease management outcomes. For type 2 diabetes, predictive models incorporating glycemic control indicators (HbA1c), inflammatory markers (IL-6, TNF-α), and metabolomic features can identify high-risk individuals 5–7 years before clinical diagnosis (39, 40). This early identification enables implementation of lifestyle interventions before irreversible pancreatic β-cell damage occurs, effectively delaying or preventing disease progression. In the field of cardiovascular disease, genetic polymorphism analysis of TNF-α and IL-6 combined with assessment of inflammatory markers and metabolic variables can identify potential risk groups for cardiovascular events among patients with chronic heart failure (41, 42).

In neurodegenerative diseases, predictive models integrating blood biomarkers such as β-amyloid and tau proteins can identify high-risk populations for Alzheimer’s disease before significant cognitive decline (43, 44). This early identification provides critical opportunities for time-sensitive interventions, enhancing treatment success probability (45). Notably, this biomarker-based early warning system represents a paradigm shift from reactive to proactive medical practice.

### 3.2 Personalized health management

Personalized health management represents the core practice of the proactive health concept. Biomarker-based predictive models facilitate the transition from population-level averages to individual precision management by providing tailored risk assessments and intervention recommendations.

#### 3.2.1 Precise prediction and intervention

Biomarker predictions based on individual genetic backgrounds and metabolic characteristics deliver more precise assessments of disease susceptibility. Machine learning methods enhance risk prediction accuracy by integrating multidimensional biomarkers with clinical data (46), particularly in specific subgroups such as diabetes patients and cancer survivors (47).

Digital twin technology facilitates the optimization of dynamic intervention strategies through real-time simulation of patient physiological data. Studies demonstrate that predictive models incorporating multiple dynamic biomarkers enable accurate patient risk stratification, establishing a more reliable foundation for clinical decision-making (48). The fundamental value of precision prediction lies in enabling targeted interventions, allowing medical resources to high-risk, patients while minimizing unnecessary medical interventions for low-risk populations (49).

#### 3.2.2 Health risk stratification

Biomarker-driven risk stratification establishes the basis for implementing differentiated health management. In metabolic disease management, risk stratification systems utilizing multiple blood markers effectively identify subgroups with different complication risks, enabling targeted intervention strategies. These systems have demonstrated efficacy in identifying diabetes subtypes associated with elevated mortality risk, supporting evidence-based precision prevention and treatment (50, 51).

The combination of cardiac biomarkers (e.g., NT-proBNP, hs-cTnT) with polygenic risk scores enhances cardiovascular risk stratification (52). This enhanced risk stratification facilitates more efficient medical resource allocation while providing patients with clearer risk information, enabling their active participation in health decision-making processes (53, 54).

#### 3.2.3 Dynamic monitoring and feedback

Periodic biomarker testing enables continuous health status monitoring. Regular assessment of marker trends facilitates early detection of abnormal changes and provides real-time feedback, supporting dynamic adjustment of intervention plans. Periodic biomarker testing enables continuous health status monitoring. Regular assessment of marker trends facilitates early detection of abnormal changes and provides real-time feedback, supporting dynamic adjustment of intervention plans. For example, wearable IoT biomarker sensors have been utilized for early disease diagnosis and continuous monitoring, significantly enhancing health management capabilities particularly in resource-limited regions (55). These devices achieve continuous sampling through microfluidic technology, providing technical support for individualized health tracking (55, 56).

Modern intelligent wearable devices incorporate continuous monitoring capabilities for multiple physiological parameters, establishing closed-loop health management systems (e.g., biofeedback-enabled wearables) when combined with specialized testing equipment. Research demonstrates that real-time heart rate monitoring through wearable devices has been implemented for stimulation decisions in closed-loop devices (57), while analysis of exercise-related electroencephalogram signals provides biomarker support for closed-loop neuroregulation (58). Such systems have significantly improved disease control outcomes, particularly in diabetes management, where closed-loop systems combining continuous glucose monitoring (CGM) with insulin pumps have achieved precise glycemic regulation (59, 60).

Dynamic monitoring enhances both disease management and long-term maintenance of healthy behaviors. Through real-time health data feedback, individuals can directly observe the immediate effects of lifestyle modifications on health indicators; for instance, biofeedback techniques in stress management can enhance positive behavioral reinforcement (61, 62). This “closed-loop” health management system embodies the fundamental concept of proactive health management-empowering individuals as active managers of their own health. The realization of personalized management ultimately depends on intelligent delivery systems that translate model outputs into actionable insights, as discussed in Section 4.4.

#### 3.2.4 Public health resource optimization

At the population health level, biomarker-driven prediction systems enhance public health resource allocation efficiency. Analysis of population-level biomarker data enables disease trend prediction and high-risk community identification, facilitating precise resource allocation. As objective indicators for disease diagnosis, treatment response prediction, and personalized medicine, biomarkers reduce medical costs and improve population health outcomes through early detection (63). Their utility as early warning systems has been validated in medical practice, with notable increases disease risk assessment applications (64). In primary prevention contexts, biomarker-supported health monitoring systems effectively identify high-risk population distributions (65).

The cornerstone of public health resource optimization lies in achieving effective population-level risk stratification. Analysis of regional biomarker data, variations enable health decision-makers to identify area-specific health risk patterns and design targeted interventions. Studies confirm that adjusting resource allocation formulas based on biomarker variations enhances both equity and efficiency in health resource distribution (66). For instance, optimizing screening service distribution based on regional disease burden characteristics through small-area health data analysis has proven effective for resource allocation (67). In urological emergencies, rapid risk stratification of urosepsis in patients with urinary calculi through urinary biomarkers allows high-risk patients to receive priority access to intensive care resources while preventing overtreatment in medium and low-risk patients, thereby reducing emergency department length of stay and healthcare costs (68).

In public health emergencies, biomarker monitoring provides early warning indicators, supporting rapid response and resource deployment. During the COVID-19 pandemic, community wastewater SARS-CoV-2 monitoring emerged as an effective tool for predicting regional epidemic trends, assisting public health departments in optimizing testing and medical resource allocation (69). In dengue-endemic regions, early warning models constructed through mosquito-borne virus genomic surveillance combined with meteorological data enable the deployment of vector control resources and mobile medical units to high-risk communities before outbreak occurrence (70, 71).

Furthermore, correlation analysis between biomarkers and environmental factors provides scientific evidence for environmental health policy development. Environmental intervention effectiveness can be evaluated through monitoring environmental exposure markers in specific populations. This data-driven approach addresses health equity issues in resource allocation (72), particularly when exposure risks demonstrate significant geographical variations, biomarker data guides policymakers in prioritizing environmental governance (73).

The successful implementation of proactive health applications requires comprehensive technical support. The following chapter details the complete technical pathway from data acquisition, and biomarker screening to model construction and optimization, providing methodological guidance for practical application implementation.

## 4 Technical framework and methodological pathway

Realizing the application value of biomarker-driven predictive models in proactive health management requires a comprehensive technical framework and methodological pathway, encompassing data acquisition, analysis, processing, model construction, evaluation, and application. This chapter systematically examines each component of this technical framework and their interconnections, providing methodological guidance for practical applications.

### 4.1 Data acquisition and preprocessing

Data acquisition and preprocessing constitute fundamental elements of the technical framework, directly influencing the quality of subsequent analysis and model construction. The primary objective of this stage involves ensuring data completeness, consistency, and reliability, establishing a high-quality foundation for subsequent analyses.

#### 4.1.1 Multi-source data integration

The integration of multi-source heterogeneous data is fundamental to developing comprehensive biomarker models. The establishment of unified data standards and interoperability frameworks facilitates seamless integration of clinical measurements, genomic information, and environmental factors (74). This integration process must address technical challenges including data format inconsistencies, terminology variations, and semantic compatibility issues (75).

Data source quality assessment and consistency verification are crucial in research practice to ensure integrated data reliability. This process involves implementing data quality scoring systems to evaluate different sources and adjust their analytical weights accordingly (76, 77). The integration process must maintain temporal consistency, ensuring proper alignment of data from various time points to support longitudinal analysis (78).

Ontology-based data integration methods serve as an effective approach, facilitating semantic mapping between diverse data sources by through shared conceptual frameworks (79). This methodology enables the creation of unified data views while maintaining original data characteristics, establishing structured foundations for subsequent analyses.

#### 4.1.2 Data quality control and standardization

Rigorous data quality control serves as the cornerstone for ensuring model performance. Multi-level quality assurance mechanisms, incorporating automated anomaly detection and standardized preprocessing workflows, should be integrated into routine components of data processing (80). Quality control protocols must encompass anomaly identification and processing, missing value assessment and imputation, duplicate record detection, and additional measures to ensure data completeness and accuracy (81).

Standardization represents a critical step in processing multi-center and multi-source data. The variation in measurement units and reference ranges, across detection platforms and laboratories often renders direct data comparison impossible (82). Standardization techniques such as Z-score normalization or percentile transformation enable conversion of diverse data sources to a unified scale (83). For high-throughput omics data exhibiting significant, specialized batch effect correction methods such as ComBat are essential (84).

The implementation of standardized data governance frameworks enhances cross-institutional data comparability and consistency, thereby improving model stability. This encompasses the establishment of data dictionaries, standard operating procedures, and quality monitoring processes to maintain consistency in data collection and processing (84).

#### 4.1.3 Privacy protection mechanisms

Clinical data processing requires adherence to strict privacy protection frameworks, that balance data utilization value with ethical requirements. Multi-level security architectures incorporate data source encryption and transmission endpoint encryption, providing continuous data protection throughout the process, exemplified by blockchain smart contract data integrity verification in multi-center medical alliances (85, 86). Attribute-based access control systems enable precise restrictions on user roles, identity characteristics, and specific operations, ensuring compliance and traceability through audit logs.

Contemporary security frameworks are designed to comply with the European Union’s General Data Protection Regulation (GDPR) requirements for sensitive medical information processing (87). Blockchain smart contracts enable regular data integrity verification, minimizing anomalies caused hardware failures, as validated on multi-center medical alliance platforms (84).

Balancing data usability with privacy protection a significant challenge. Advanced technologies including differential privacy and federated learning enable collaborative analysis and model training without raw data sharing (88, 89). These innovations create new opportunities for multi-center collaborative research while safeguarding patient privacy and data sovereignty.

The data acquisition and preprocessing phase establishes the foundation for subsequent biomarker screening and model development. High-quality, standardized, and secure data constitutes a prerequisite for achieving accurate predictions and effective clinical applications. The following section explores methods for screening and validating clinically valuable biomarkers from this data.

### 4.2 Biomarker screening and validation

Biomarker screening and validation is a key link connecting data and models, with the goal of identifying indicator combinations with clinical predictive value from massive data. This process requires combining statistical methods, machine learning techniques, and biological knowledge to ensure that the selected biomarkers have statistical significance, biological rationality, and clinical practicality.

#### 4.2.1 Application of machine learning and statistical methods

The identification, of clinically valuable biomarkers from multi-omics data represents a central challenge in precision medicine. While traditional statistical methods offer robustness, they encounter limitations in processing high-dimensional data and non-linear relationships (90). Recent machine learning advances, particularly ensemble algorithms such as Extreme Gradient Boosting (XGBoost) and Deep Neural Networks (DNNs), have enhanced biomarker screening efficiency through automatic capture of non-linear interactions and hierarchical feature expressions (91, 92). Wrapper feature selection techniques utilizing genetic algorithms can evaluate multiple feature combinations to optimize model performance in supervised tasks such as cancer classification (93). These methodologies demonstrate superior capabilities in feature selection and pattern recognition compared to conventional approaches.

The integration of machine learning with statistical methods addresses the limitations of conventional approaches in identifying non-linear relationships. Evidence indicates that this combined approach enhances screening efficiency and accuracy while maintaining statistical rigor, generating novel insights for biomarker research (94). The practical implementation typically involves statistical methods for initial screening, followed by machine learning techniques for detailed analysis, and subsequent validation through biological knowledge assessment. Research demonstrates that these integrated approaches improve Area Under the Curve (AUC) values by 12–18% in survival prediction and disease classification compared to individual methods (95, 96).

Data preprocessing plays a vital role in ensuring reliable analysis and model performance. Box plots and interquartile range (IQR) methods effectively handle outliers, while missing data managed through Multiple Imputation by Chained Equations (MICE) maintains sample integrity (12, 90). For multi-center studies with batch effects, the ComBat normalization algorithm employs a Bayesian framework to address platform biases, enhancing cross-dataset biomarker comparability by over 30% (97).

The optimization of screening strategies necessitates a balance between statistical power and computational efficiency. For high-dimensional data analysis, two-stage screening approaches demonstrate superior effectiveness: initially employing rapid algorithms (e.g., LASSO regularization or random forest importance scoring) for preliminary screening (98), followed by comprehensive evaluations of candidate features, including statistical significance testing, stability analysis, and biological pathway enrichment analysis (99). This approach reduced model computation time by 58% in renal transplant cardiovascular risk prediction while maintaining 95% prediction accuracy (100). The integration of these computational approaches with biological context is particularly crucial in multi-omics research, where heterogeneous data layers demand unified analytical frameworks, as discussed below.

#### 4.2.2 Multi-omics integration research design

Multi-omics research design facilitates comprehensive understanding of disease mechanisms. While single omics data (e.g., genomics) provides substantial information, it often fails to capture the complete complexity of diseases. The integration of multi-level biological information, including genotype, expression profiles, protein levels, and metabolites, enables the construction of more comprehensive molecular disease maps (101).

The combination of proteomics, metabolomics, and genomics data yields more comprehensive molecular characteristics. This integrated approach enables the identification of essential biomarkers and pathways that remain undetectable through single omics methods (102). Various integration strategies are applicable, including early integration (merging data at the feature level), intermediate integration (merging results at the model level), and late integration (merging predictions at the decision level).

Multi-omics integration presents technical challenges including data dimension imbalance, feature scale differences, and data completeness issues. Specialized integration algorithms, such as multi-view learning and tensor decomposition methods, effectively address these challenges by processing heterogeneous data sources and extract complementary information (103). During validation, careful consideration of the incremental value of integrated models compared to single omics models, ensures substantial improvements in predictive performance.

Multi-omics research design requires consideration of cost-effectiveness balance. Although comprehensive multi-omics analysis generates extensive information, clinical implementation remains costly and complex (104). Selecting optimal omics combinations with maximum incremental predictive value achieves optimal cost-effectiveness ratios for specific applications. For routine clinical applications, developing simplified models incorporating select high-value biomarkers based on previous research enhances clinical feasibility (105).

Systematic biomarker screening and validation enables the identification of clinically predictive indicator combinations, establishing a foundation for subsequent model construction. These validated biomarkers demonstrate both statistical predictive power and biological disease mechanisms, facilitating the translation from data to clinical insights.

### 4.3 Model development and optimization

Model development and optimization is the core component of the technical framework, transforming screened biomarkers into predictive tools for clinical decision-making. This stage requires selecting appropriate algorithms, optimizing model structure and parameters, validating model performance, and ensuring clinical applicability of the model.

#### 4.3.1 Multifactor prediction model construction

In contemporary medical research, multifactor predictive models have largely superseded univariate analysis as primary tools for disease risk assessment. Biomarker screening provides fundamental support for model development (106), but comprehensive disease mechanism analysis requires integration of exogenous variables (e.g., environmental exposures and behavioral patterns). Model selection should align with data characteristics and research objectives: logistic regression offers high interpretability for quantifying biomarker-disease associations (107), while ensemble methods (e.g., random forests) provide robust performance in capturing complex nonlinear patterns (108).

Deep learning enhances high-dimensional heterogeneous data processing through multiple non-linear transformations to extract potential feature patterns (109). However, its inherent “black box” nature constrains clinical translation (110). The introduction of enhanced interpretability techniques (e.g., attention mechanisms and SHAP value frameworks) addresses this limitation, balancing predictive accuracy with model transparency (111). This technological integration establishes new pathways for clinical implementation of predictive models.

Hierarchical model building strategies demonstrate superior outcomes, by first assessing foundation models based on clinical and routine biochemical indicators, then evaluating the incremental predictive value of novel biomarkers (106). This methodology quantifies the contribution of new markers while ensuring models maintain clinical utility. For complex diseases, stratified modeling approaches, which develop specialized predictive models for distinct disease subtypes or risk levels, effectively enhance prediction accuracy.

#### 4.3.2 Feature engineering

The predictive performance of multifactor models relies on the relevance and representativeness of selected features. As the foundation of model development, feature engineering significantly influences model accuracy and generalizability through the selection, transformation, and creation of variables to optimize attribute representation. Dimensionality reduction techniques (e.g., PCA, t-SNE) simplify complex biomarker data while preserving critical information (112).

In clinical applications, feature interpretability and stability are essential considerations, making feature combinations with clear biological significance preferable. Domain knowledge is instrumental in feature engineering, guiding the development of clinically meaningful derived features, such as establishing “intervention measures” and “orthotic device” related feature sets through medical expert knowledge (113), or developing dynamic indicators such as biomarker ratios and rates of change based on pathological progression mechanisms (114).

The synthesis of clinical experience and algorithmic feature selection optimally identifies variable combinations with biological significance. For instance, in cardiovascular risk prediction, combining 7 imaging features selected by LASSO regression with 4 clinical features achieved an AUC of 0.848 (115); while in sepsis prediction, incorporating hemodynamic fluctuation indicators with temporal trend features through random forest algorithms enhanced the model’s capacity to capture pathological dynamic changes (116). These dynamic features provide predictive information beyond traditional static indicators by reflecting longitudinal change patterns of biomarkers (e.g., cerebral blood volume fluctuations, metabolite time series fluctuations).

Feature engineering must address the feasibility of practical clinical applications. Optimal feature combinations should balance predictive performance, measurement cost, and operational complexity. Studies indicate that simplified models integrating routine examination indicators (e.g., prostate-specific antigen density) with radiomics features reduced feature dimensions from 107 to 8 while maintaining an AUC of 0.774 (117). In implementation, developing predictive models of varying complexity, from simplified versions with routine examination indicators to advanced versions incorporating multi-omics data, enables adaptation to different application scenarios and resource constraints.

#### 4.3.3 Model validation and evaluation

Rigorous validation frameworks are essential for ensuring model reliability in real applications. Multi-level validation strategies, encompassing internal cross-validation, external independent cohort validation, and robustness testing for different populations, represent standard practices in modern model evaluation. Internal validation typically employs k-fold cross-validation (118) or bootstrapping to assess model stability on training data; external validation tests model generalizability in new populations or different clinical environments (119), a critical step in determining the model’s true value.

Comprehensive evaluation frameworks should incorporate multiple performance metrics to thoroughly assess the model’s predictive capability, stability, and clinical value. For classification tasks, beyond traditional AUC, sensitivity, specificity, and positive predictive value warrant examination; for prognosis prediction, calibration (consistency between predicted probabilities and actual event rates) and discriminative ability (e.g., C-index) require evaluation (120, 121). Decision Curve Analysis (DCA) has emerged as a vital tool for evaluating clinical value, quantifying net benefits at different decision thresholds (122).

Transparency and completeness in the validation process are crucial. In paper publications and clinical applications, validation methods, population characteristics, and all performance metrics require detailed reporting, enabling other researchers and clinical users to accurately assess the model’s applicable scope and limitations. Additionally, public sharing of model code and validation datasets promotes research transparency and reproducibility.

#### 4.3.4 Clinical utility evaluation

The ultimate value of predictive models resides in their clinical utility. Tools such as decision curve analysis and clinical impact graphs quantify the net benefit of models at different decision thresholds. These methods evaluate the value of models in actual clinical scenarios, considering the relative costs of false positives and false negatives, providing a foundation for selecting decision thresholds (123).

Research demonstrates that integrating prediction results with existing clinical risk scores substantially increases physicians’ trust in model outputs (124). Developing decision support tools compatible with existing clinical workflows, enabling model predictions to integrate seamlessly into daily clinical practice, represents an effective strategy for improving clinical acceptance (125). Model outputs should be presented in intuitive and comprehensible forms, such as risk classifications, risk percentiles, or comparisons with reference populations, enabling informed decision-making by clinicians and patients.

Clinical utility evaluation should consider implementation costs and operational complexity. High-performance but complex and expensive models may face limitations in practical applications (126), necessitating balance between so performance and feasibility during model development. Early consideration of model clinical pathway integration strategies ensures effective translation of technological innovation into clinical value.

#### 4.3.5 Iterative model optimization

Prediction model development is an iterative process necessitating continuous refinement based on evolving clinical needs, emerging data, and performance feedback from real-world deployment. Optimization cycles involve re-evaluating model architecture, retraining with updated datasets, adjusting hyperparameters, and incorporating novel biomarkers or features to enhance predictive accuracy, stability, and clinical utility over time (127, 128). Moreover, establishing mechanisms for regular model performance monitoring, feedback collection from end-users (clinicians, patients), and scheduled re-validation is critical, particularly as clinical practices evolve or population characteristics shift. This ongoing process ensures the model remains relevant, reliable, and effective in supporting clinical decision-making within the dynamic healthcare environment.

Following model development and optimization, the critical next phase involves effectively delivering prediction results to end users to realize their clinical value.

### 4.4 Model prediction results delivery

The effectiveness of model predictions ultimately depends on how results are communicated and used. Prediction results delivery is the key link in transforming model outputs into actionable information, requiring consideration of user needs, information presentation methods, and decision support functions.

#### 4.4.1 Intelligent delivery system construction

Intelligent results delivery systems should adapt information display methods according to specific user roles and clinical contexts. For clinicians, the system provides comprehensive risk assessment results, including key risk factors, recommended interventions, and expected outcomes (129); for patients, it delivers accessible risk information and personalized health recommendations (130); for health management organizations, it generates population-level risk analysis reports to support resource allocation decisions (131).

Delivery mechanisms incorporating decision support functions and interpretability tools enhance clinical acceptance. These include providing evidence links, explaining primary predictive factors, comparing potential outcomes of different intervention strategies, and offering personalized recommendations based on historical decision patterns (132–134). Results delivery should integrate risk explanation with actionable intervention recommendations to maximize clinical utility.

From a technical perspective, delivery systems should facilitate multi-platform integration and real-time updates. Contemporary delivery architectures typically encompass web applications, mobile applications, and electronic health record system integration, ensuring accessibility across various environments (135). Moreover, systems should incorporate configurable alert mechanisms that automatically generate notifications based on predicted risk levels and time sensitivity, enabling timely intervention for high-risk cases (136).

**Figure 2:**
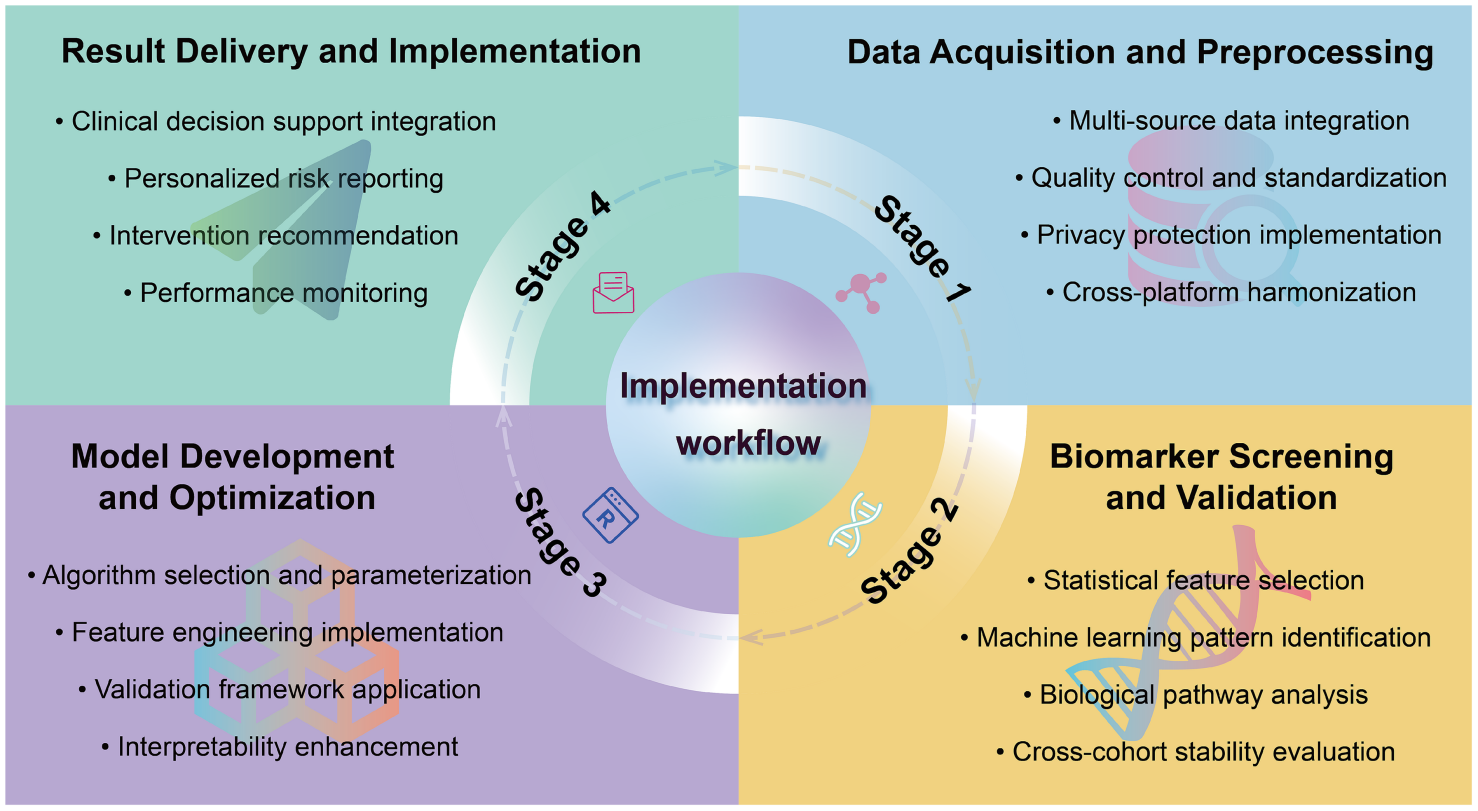
A data-driven framework for health management: from biomarker discovery to personalized health implementation. The diagram depicts the cyclical process of: (I) Data Acquisition and Preprocessing (show in blue); (II) Biomarker Screening and Validation(show in yellow); (III) Model Development and Optimization(show in purple); (IV) Result Delivery and Implementation(show in green). Each stage contains essential components for translating biomarker discovery into clinical application, with implementation workflow at the center.

Through a comprehensive technical framework and methodological pathway, biomarker-driven predictive models establish a closed loop from data acquisition and analysis to clinical application, achieving proactive health management objectives (Figure 2). This integrated framework illustrates how the systematic processing of biomarker data through standardized analytical pathways enables transition from reactive medical interventions to proactive health monitoring and personalized risk assessment, representing a paradigm shift in clinical practice methodology. Nevertheless, this process faces multiple challenges requiring systematic response strategies, which the next chapter examines in detail.

## 5 Data quality and standardization challenges

The challenges facing biomarker-based predictive models span the entire pipeline from data to clinical implementation: data quality and standardization issues constitute fundamental barriers at the foundational level; limited model generalizability restricts the broad applicability of these technologies; difficulties in clinical translation of biomarkers impede the transformation of research findings into clinical practice; while public acceptance and resource constraints affect the ultimate implementation effectiveness. These challenges form a multi-layered problem system from technical to social dimensions, requiring systematic solutions.

### 5.1 Data quality and standardization

Challenges: Clinical test data originates from multiple sources, lacking standardized collection and storage protocols (137). Variations in testing equipment parameters and operational standards create systematic biases in cross-institutional indicators (138). Manual input errors and heterogeneous system compatibility issues may compromise data integrity, affecting the reliability and comparability of subsequent analyses (139). This data heterogeneity constrains predictive model optimization, creating structural barriers to multi-scenario applicability (140).

Solutions: Implement quality control systems and standardize data governance from the source, conducting regular equipment calibration and enhancing operator training. In data processing, establish traceable cleaning mechanisms and standardized outlier handling protocols (141). Systematically standardize the entire data lifecycle management process according to international clinical laboratory quality management systems (142). This includes establishing unified data collection and coding standards, implementing standardized medical terminology systems (e.g., SNOMED CT or LOINC) (140), and deploying automated data quality monitoring processes.

These measures will enhance data quality, establish a robust foundation for model construction, and strengthen prediction system reliability (143). Meanwhile, developing multi-center data collaboration networks and sharing standardized data collection and processing methods can accelerate high-quality health data accumulation (144), providing adequate training samples for accurate predictive models.

Additionally, we recommend developing intelligent data cleaning and standardization tools for automatic identification and processing of common data issues, including outlier detection, unit conversion, and missing value handling. These tools should be adaptable to accommodate the requirements of various medical environments and data types, while maintaining comprehensive records of the processing, to ensure transparency and traceability of data processing (145).

### 5.2 Model generalizability

Challenges: Current predictive models frequently experience performance degradation in “laboratory-to-clinical” scenarios, primarily due to limitations in training data regarding geographic, age, and racial diversity (146). This insufficient data representativeness impedes the model’s capacity to capture complex population differences, reducing its adaptability in clinical healthcare (147). Models demonstrate performance deterioration in real-world clinical practice implementation; this “laboratory-to-reality” performance gap represents a major obstacle to the implementation of precision medicine (128, 148).

Solutions: Enhancement of model generalizability necessitates constructing training datasets with greater ecological validity. Multi-center collaborative collection of health data encompassing geographic/demographic/environmental variations can strengthen model generalization capabilities through the integration of diverse feature sets (146). In model design, we recommend adopting domain adaptation and transfer learning techniques to enable models to better adapt to feature distributions in new populations. Incremental learning strategies facilitate model updates with new data without complete retraining (149), which is essential for dynamically adapting to changes in clinical environments.

In model validation, we recommend combining independent cohort validation with real-world data testing. Robustness testing that considers dynamic environmental factors and individual differences effectively identifies models with clinical utility (150, 151). This multidimensional validation system provides a scientific basis for model deployment, while identifying the model’s applicable scope and limitations, offering clear guidance for clinical use.

Furthermore, we advocate developing integrated and adaptive model architectures capable of automatically adjusting prediction strategies based on different population characteristics. For instance, utilizing meta-learning frameworks to build models that rapidly adapt to new populations, or adopting stratified model strategies to train specialized models for different population subgroups and then integrating results through ensemble methods. This approach better adapts to population heterogeneity while maintaining overall prediction accuracy (152).

### 5.3 Difficulty of clinical translation of biomarkers

Challenges: A substantial gap exists between basic biomarker research and clinical applications, with detection performance variability and disease heterogeneity serving as key bottlenecks (153). Although high-throughput detection enhances sensitivity, its high cost and complex operations restrict widespread use (154). Moreover, clinical validation requires large-scale, prospective longitudinal cohorts (155), further decelerating the translation process. Clinical application of novel biomarkers faces extended processes of regulatory approval (156) and inclusion in clinical guidelines (157), impeding the timely delivery of innovative results to patients.

Solutions: Addressing this challenge requires establishing “basic-clinical” feedback mechanisms. Systematic evaluation of marker predictive performance in various clinical scenarios through multi-center trials and establishment of automated testing platforms balances accuracy and cost. This evaluation should follow unified methodological standards and reporting guidelines (158), ensuring result comparability and reliability.

Formulating standardized clinical guidelines and dynamic evaluation systems is essential for biomarker standardization. We recommend establishing biomarker evaluation alliances (157), integrating resources from academic institutions, medical centers, and industry (158) accelerates the validation and translation of high-value markers. Meanwhile, developing simplified testing technologies and point-of-care devices reduces the cost and complexity of high-value biomarker detection, enhancing suitability for routine clinical applications.

We believe that constructing a complete translation pipeline from discovery to application, including early clinical validation, commercialization pathway planning, and regulatory strategy formulation (159), can expedite the clinical translation of biomarkers. This process, close collaboration between academia and industry for efficient transformation of scientific discoveries into clinical testing products. Establishing biomarker research and development sharing platforms promotes open sharing of data, methods, and resources helping avoid duplication and accelerate innovation.

### 5.4 Public acceptance of artificial intelligence and big data

Challenges: While technological safeguards (Section 4.1.3) address data security, public acceptance requires additional sociotechnical considerations, including algorithmic transparency and ethical governance. Advanced medical AI applications raise concerns regarding algorithmic interpretability and privacy security. Limited trust in prediction results among healthcare providers and patients stems from insufficient technological transparency and risk communication (160). Vulnerabilities in health data lifecycle management, particularly leakage risks, intensify public skepticism and concern (161). Inadequate consent mechanisms in data collection processes and concerns about health data usage impede public acceptance of AI-based health solutions (162).

Solutions: Establishing technological trust requires multidimensional public engagement. Creating open technology exchange platforms to explain AI decision logic and uncertainty enhances prediction result transparency for healthcare providers and patients. This includes developing visualization tools to display key predictive factors and uncertainties, and providing user-friendly explanation systems that translate complex model decisions into comprehensible language (163).

For data security, we recommend technologies such as differential privacy and federated learning, and implementing layered authorization and traceability mechanisms. These technologies enable model training and prediction while protecting data privacy, minimizing data sharing (164). Establishing robust data governance frameworks, including clear data usage policies, transparent consent processes, and comprehensive security measures, forms the foundation for building public trust.

We propose that comprehensive technological ethics governance frameworks can enhance public acceptance as a positive catalyst for implementation. This involves establishing multidisciplinary ethics committees to oversee the development and deployment of AI systems, ensuring technological innovation prioritizes patient welfare (160). Conducting systematic public education and engagement initiatives to explain the capabilities and limitations of AI in healthcare helps foster public understanding and acceptance of these emerging technologies.

Additionally, implementing clear responsibility and accountability mechanisms at the policy level clarifies the obligations of various stakeholders in data use and AI deployment. Well-defined regulatory frameworks protect patient rights while providing clear guidance for technological innovation, fostering sustainable industry development (165).

### 5.5 Resource and cost issues

Challenges: Developing high-performance predictive models requires integrating expertise from medicine, computer science, and biology, but the scarcity of interdisciplinary professionals presents a major constraint (166). Model development requires terabyte-scale data storage and high-performance computing, creating substantial hardware demands, while ongoing R&D investment strains small and medium-sized organizations (167, 168). In the large-scale clinical implementation, costs of iterative device upgrades and network infrastructure maintenance seriously impact resource allocation (169). These resource constraints affect both technological development and deployment in resource-limited settings (170).

Solutions: Developing comprehensive talent cultivation systems and industry-academia-research collaboration mechanisms is essential for addressing resource limitations. Higher education institutions can nurture multidisciplinary professionals with both clinical expertise and algorithm development capabilities through interdisciplinary laboratories (166). Meanwhile, implementing data science training programs for practicing healthcare professionals enhances the technical capabilities of existing medical teams effectively addressing immediate talent shortages (171).

Establishing data resource sharing and technology collaboration platforms between healthcare institutions, research institutes, and enterprises can minimize translation costs. Such partnerships can leverage the distinctive resources of different institutions, prevent redundant investments, and expedite the translation of innovations. For example, healthcare institutions provide clinical data and application scenarios, research institutions contribute algorithms and analytical methods, and enterprises deliver technological implementation and productization support, creating a synergistic research ecosystem (170, 172).

Developing intelligent medical technology training programs for practicing personnel can enhance the technological integration capabilities of existing teams, facilitating predictive model adoption. This includes developing modular and customizable learning resources, enabling healthcare professionals to learn according to their needs and schedules (173). Meanwhile, developing “technical assistant” systems to support clinicians using complex prediction tools effectively reduces technological usage barriers (174).

For resource optimization, we recommend implementing architectures that combine cloud computing and edge computing to balance performance and cost. Cloud computing offers flexible computational resources that adjust dynamically to demands, avoiding substantial fixed asset investments (175); edge computing processes certain data and computational tasks locally, reducing network bandwidth requirements and latency (167). For resource-constrained environments, developing efficient algorithms and model compression techniques enables prediction systems to operate effectively on standard hardware.

Through systematic approaches to these challenges, biomarker-based predictive models can overcome current development constraints and more effectively support proactive health management objectives. Table 3 summarizes the primary challenges facing biomarker predictive models and their solutions, highlighting the multifaceted efforts required to advance technology from laboratory to clinical application. This structured framework illustrates why successful implementation requires coordinated efforts across technical, clinical, and social domains to deliver the potential benefits of predictive models in real-world healthcare settings. The next chapter summarize the main findings and contributions of this research and look toward future development directions.

**Table 3:** Challenges and solution strategies for clinical translation of biomarker predictive models.

Challenge dimension	Key issues	Core solutions	Implementation examples
Data quality and standardization	Cross-platform heterogeneity, batch effects	Unified data governance framework, standardized biorepositories	Global biomarker reference standards
Model generalizability	Insufficient population diversity, temporal stability issues	Multi-center validation, transfer learning methods	Diverse validation cohorts
Clinical translation	Basic-clinical gap, regulatory uncertainties	Simplified detection technologies, standardized evaluation systems	Point-of-care testing development
Public acceptance	Privacy concerns, transparency issues	Privacy-preserving computing, explainable AI approaches	Federated learning implementation
Resource limitations	Interdisciplinary talent gaps, infrastructure challenges	Talent development programs, tiered implementation strategies	University-hospital collaborations
Ethical considerations	Health inequality risks, algorithmic bias	Equity assessment frameworks, inclusive design methodologies	Ethical impact assessments

AI, Artificial Intelligence.

## 6 Conclusion and outlook

### 6.1 Application prospects and remaining challenges

Biomarker-based predictive models advance proactive health management by integrating biomarker data with computational technologies. These models demonstrate potential in: (1) Early disease detection at pre-clinical stages; (2) Dynamic health monitoring with real-time assessment; (3) Evidence-based healthcare resource optimization.

However, challenges persist: data heterogeneity, limited model generalizability, high costs, and public trust concerns. Technological advancements including federated learning, differential privacy techniques, simplified testing technologies, and improved model explanation tools will progressively address these barriers.

### 6.2 Research contributions and innovation value

This review’s contributions include: (1) Establishing a comprehensive framework connecting biomarker discovery with clinical application; (2) Presenting viable solutions for key implementation challenges through data governance frameworks and validation strategies; (3) Investigating the interplay between technological advancement and ethical considerations; (4) Expanding biomarker-driven frameworks to public health decision-making.

### 6.3 Future research directions

Future research should prioritize: expanding to rare disease markers and dynamic health indicators; deepening multi-omics integration through systematic analysis of genomic, epigenomic, transcriptomic, proteomic, and metabolomic data; conducting longitudinal studies covering the entire life cycle; developing edge computing and lightweight algorithms for resource-constrained environments; and strengthening cross-collaboration between medicine, computer science, and social sciences.

### 6.4 Summary and vision

This review establishes a multidisciplinary framework for advancing biomarker-driven predictive models in proactive health management, addressing critical barriers in data standardization, model interpretability, and clinical implementation.

Biomarker-based predictive models represent a paradigm shift from reactive medicine to proactive prevention. Through integration of multidimensional biomarker data with advanced computational methodologies, these systems enable early disease detection, risk stratification, and personalized interventions—foundational elements for evidence-based health management. The technical framework proposed herein provides structured guidance for real-world healthcare applications.

This prevention-centered paradigm represents a viable approach for enhancing population health outcomes. As technological innovation advances and interdisciplinary collaboration strengthens, biomarker-driven predictive models will assume increasingly significant roles in health management, contributing substantially to disease prevention initiatives. This transition requires technological advancement alongside appropriate regulatory frameworks, ethical considerations, and societal engagement across medical, technological, and societal domains.
